# Smoking

**Published:** 1879-02

**Authors:** 


SMOKING.—Mr. Parton, who devotes his life to literary labor, and who was himself a smoker once, says :

" The vast majority of smokers—seven out of every ten—can, without the least danger or inconvenience, cease smoking totally and for ever. I was myself a smoker for thirty years, but I am now free ; I can work better and longer than before; I have less headache ; I have a better opinion of myself ; I enjoy exercise more, and step out much more vigorously ; my room is cleaner ; I think I am better tempered, as well as more cheerful and satisfied ; it did not pay to smoke, but most decidedly it pays to stop smoking."

There is a better and more manly way than this : omit the liquor, have the moral courage to plant your foot down and say " I'll never smoke again," and let that be that be end of it ; he who leans upon brandy for any thing leans upon a spear which, in the breaking, may pierce him to the heart.

